# Automated pulmonary nodule classification from low-dose CT images using ERBNet: an ensemble learning approach

**DOI:** 10.1007/s11517-025-03358-2

**Published:** 2025-04-15

**Authors:** Yashar Ahmadyar, Alireza Kamali-Asl, Rezvan Samimi, Hossein Arabi, Habib Zaidi

**Affiliations:** 1 Department of Medical Radiation Engineering, Shahid Beheshti University, Tehran, Iran; 2 Division of Nuclear Medicine & Molecular Imaging, Geneva University Hospital, CH- 1211 Geneva, Switzerland; 3 Department of Nuclear Medicine and Molecular Imaging, University of Groningen, University Medical Center Groningen, Groningen, Netherlands; 4 Department of Nuclear Medicine, University of Southern Denmark, 500 Odense, Denmark; 5 University Research and Innovation Center, Óbuda University, Budapest, Hungary

**Keywords:** Computed tomography, Lung cancer, Classification, Low dose, Deep learning

## Abstract

The aim of this study was to develop a deep learning method for analyzing CT images with varying doses and qualities, aiming to categorize lung lesions into nodules and non-nodules. This study utilized the lung nodule analysis 2016 challenge dataset. Different low-dose CT (LDCT) images, including 10%, 20%, 40%, and 60% levels, were generated from the full-dose CT (FDCT) images. Five different 3D convolutional networks were developed to classify lung nodules from LDCT and reference FDCT images. The models were evaluated using 400 nodule and 400 non-nodule samples. An ensemble model was also developed to achieve a generalizable model across different dose levels. The model achieved an accuracy of 97.0% for nodule classification on FDCT images. However, the model exhibited relatively poor performance (60% accuracy) on LDCT images, indicating that dedicated models should be developed for each low-dose level. Dedicated models for handling LDCT led to dramatic increases in the accuracy of nodule classification. The dedicated low-dose models achieved a nodule classification accuracy of 90.0%, 91.1%, 92.7%, and 93.8% for 10%, 20%, 40%, and 60% of FDCT images, respectively. The accuracy of the deep learning models decreased gradually by almost 7% as LDCT images proceeded from 100 to 10%. However, the ensemble model led to an accuracy of 95.0% when tested on a combination of various dose levels. We presented an ensemble 3D CNN classifier for lesion classification, utilizing both LDCT and FDCT images. This model is able to analyze a combination of CT images with different dose levels and image qualities.

**Graphical abstract:**

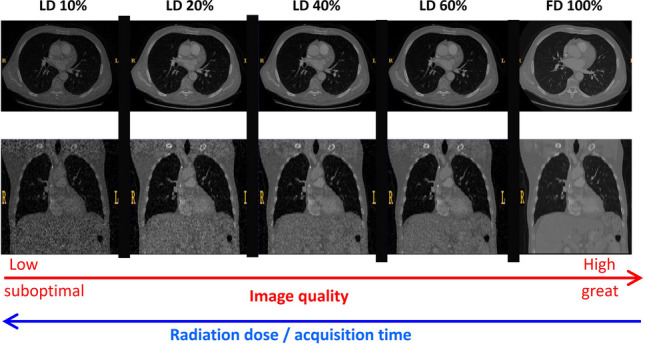

**Supplementary Information:**

The online version contains supplementary material available at 10.1007/s11517-025-03358-2.

## Introduction

Several morphological characteristics of pulmonary nodules have been shown to influence their potential malignancy. This includes size, shape, and growth. To differentiate benign from malignant nodules and avoid further costly examinations, conventional imaging techniques can be used to evaluate the morphologic characteristics of pulmonary nodules [1]. In this regard, early low-dose CT (LDCT) screening is highly recommended for lung cancer detection [2].

Recent studies have shown that LDCT-based lung cancer screening leads to statistically significant drops in lung cancer mortality in high-risk populations [3, 4]. Hence, the protection of patients from excessive radiation exposure is of the utmost importance during screening procedures. Due to low cost and low radiation exposure, LDCT imaging is frequently used to detect lung nodules. Nevertheless, it is crucial to note that LDCT, especially in screening procedures, may potentially play a role in cancer development, as suggested by certain studies [5, 6]. It is, therefore, highly desirable to utilize techniques that deliver a lower radiation dose. However, lowering radiation dose in LDCT often results in increased image noise and reduced image quality, which can affect diagnostic accuracy. It is, therefore, highly desirable to utilize techniques that deliver a lower radiation dose, like early screening using chest radiography. However, previous studies demonstrated that chest radiographs would miss almost 77% of cancer cases that could be diagnosed by LDCT [7–12].

Low-dose imaging would result in remarkably increased noise levels in CT images, which would limit the clinical value of CT imaging and the accuracy of nodule identification. Furthermore, LDCT presents significant challenges in achieving consistent image quality across different scanners, protocols, and medical centers. Variability arises due to differences in scanner hardware, such as detector technology, tube current, and voltage settings, which impact the noise levels and resolution of the images. Additionally, imaging protocols can vary widely between centers, with factors, such as slice thickness, reconstruction algorithms, and scan parameters, differing based on institutional preferences or equipment capabilities. These inconsistencies make it difficult to standardize LDCT images, leading to variations in diagnostic accuracy and complicating the development of universal image processing algorithms. Hence, nodule diagnosis becomes a challenging task due to the above-mentioned issues. Recent studies have focused on reducing statistical noise in low-dose computed tomography (LDCT) imaging by employing deep learning models to transform LDCT images into their corresponding full-dose, denoised versions. While these advancements have enhanced image quality, there remains a notable gap in research concerning pulmonary nodule detection across varying low-dose CT levels. Our study pioneers in this domain by introducing ERBNet, an ensemble learning approach designed for automated pulmonary nodule classification using LDCT images at different dose levels. This work represents the first comprehensive investigation into nodule detection and classification tailored to the nuances of LDCT imaging across different levels of low-dose CT imaging [13–16].

Another challenge is the time-consuming process of classifying nodules and non-nodules for experts, which is subject to variability depending on their experience and expertise. Deep convolutional neural networks (DCNNs) are able to detect malignant lung nodules by identifying a number of complex image features (sometimes combined with clinical factors) [17]. In this regard, several studies have attempted to detect lung abnormalities and risky nodules in CT images using DCNNs [18–24]. The size, morphology, and texture of lesions are critical factors in distinguishing between nodules and non-nodules. Additionally, these lesions exhibit patterns in imaging data that challenge human comprehension. However, deep learning algorithms, capable of examining every pixel in an image, can identify extremely subtle features that may escape human observation.

Regarding the classification of lung nodules and non-nodules on LDCT images, recent studies developed methodologies to classify malignant and benign nodules [25–31]. In this study, we first aim to investigate the effect of lower-quality LDCT images on the performance of DCNNs for classification of lung nodules and non-nodules by simulating reduced-dose CT scans. This simulation mimics the goal of decreasing LDCT dose to minimize tissue damage, while also accounting for variations in image quality caused by different scanners and protocols. Second, we aim to develop a lightweight neural network to assist physicians in the classification considering the lower quality typical of conventional LDCT. Moreover, we aimed to present a generalized model applicable across various levels of dose and image qualities.

Acknowledging the importance of thin section CT evaluations from different views [32], we trained our model on 3D images. This approach allows for the analysis of lesion features and their correlations in different directions and views, enhancing the comprehensive assessment of lung nodules. Lesion classification can be performed after denoising LDCT images. However, the aim of this work is not to develop a denoising framework for nodule detection, as the classification task can be much easier for DCNNs in terms of accuracy and computational cost. The process of identifying nodule candidates can be automated using detection deep algorithms [24] or performed manually by a radiologist. Subsequently, the suspicious regions are subjected to evaluation within our proposed model, which is capable of analyzing the region of interest regardless of the quality or dosage of the CT image. This model then provides an assessment regarding the presence or absence of nodules, facilitating a secondary review.

## Materials and methods

### Study protocol and objectives

**Fig. 1:**
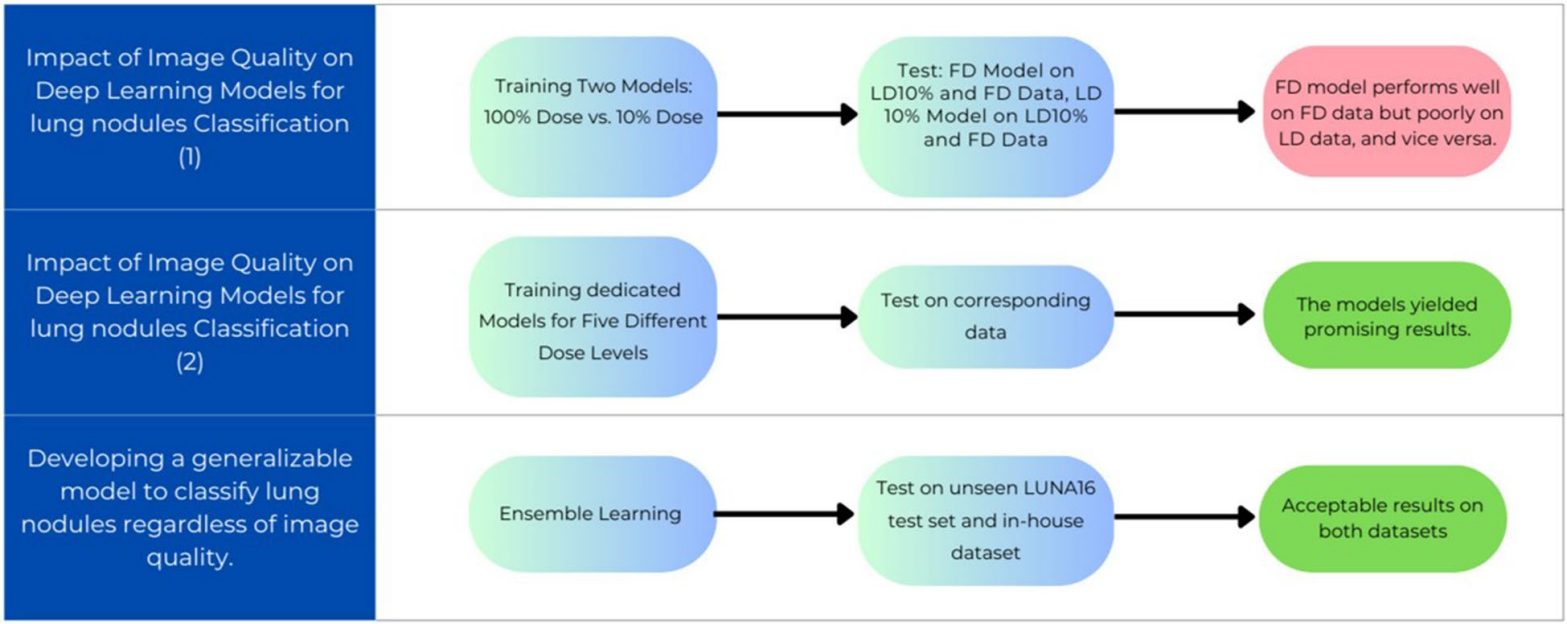
Overview of the study protocol

The main objective of this study is to classify lung nodules and non-nodules from low-dose CT images. First, four levels of LDCT images were generated using Beer-Lambert’s law; then patches of 64 × 64 × 64 voxels were extracted and fed into a CNN-based classifier. A lightweight deep learning architecture was developed, and model training was performed using five different dose levels for nodule classification. Different levels of low-dose images along with full-dose images were separately tested for model evaluation. The performance of the model trained on the full-dose CT images (full-dose model) was assessed for the different LDCT images as input. Moreover, the dedicated models trained for a specific low-dose level were evaluated using the same LDCT images (Fig. 1).

Finally, we developed an ensemble model using weighted averaging combining the predictions of five models trained on different dose levels. This ensemble approach enables to leverage the strengths of each individual model and generates more robust and accurate predictions for diverse dose levels in the dataset. The ensemble model was developed using weighted averaging of individual models trained on different dose levels. In this approach, each model contributes differently to the final ensemble, with its influence determined by performance-based weights. These weights were optimized by evaluating the models 20,000 times using 20,000 randomly generated vectors from a Dirichlet distribution to identify the best-performing combination on a blind test dataset.

### Dataset and preprocessing

The deep learning models used in this study were developed using 888 clinical CT images from the Lung Nodule Analysis 2016 (LUNA16) dataset (available at: https://luna16.grand-challenge.org/Data/). This dataset is a curated subset of the LIDC-IDRI dataset [33], which consists of thoracic CT images annotated by multiple expert radiologists. The LUNA16 dataset contains 512 × 512 × 100–500 voxel CT scans and provides detailed nodule annotations based on a multi-radiologist review process. Each CT image was independently reviewed by four expert radiologists, who marked lesions as nodules or non-nodules. This annotation process followed a two-phase approach:

- *Blinded read phase:* Each radiologist independently marked lesions without access to others’ markings.
- *Unblinded read phase:* Radiologists reviewed their own and others’ annotations before finalizing their classifications.

To ensure robust labeling, a lesion was regarded as a clinically significant nodule if at least three out of four radiologists agreed that it met the criteria for being classified as a nodule. The dataset distinguishes three categories of pulmonary lesions:

- *Nodules* ≥ *3 mm:* Lesions that were explicitly outlined and assessed for their clinical relevance.
- *Nodules* < *3 mm:* Small lesions that were not necessarily excluded from analysis but were only recorded with center-of-mass locations instead of full segmentation outlines. These nodules were considered of lower clinical significance, given current diagnostic guidelines.
- *Non-nodules:* A broad category that includes other pulmonary abnormalities that could be misinterpreted as nodules, such as vascular structures, calcified granulomas, fibrotic changes, and inflammatory areas.

Contrary to a strict size-based classification, nodule and non-nodule differentiation was not determined solely by the 3-mm threshold. Instead, radiologists evaluated lesion characteristics, such as shape, texture, margin sharpness, and anatomical location, to differentiate between nodules and non-nodules. This approach ensured that the dataset accounted for a wide range of confounding pulmonary structures, including inflammatory areas and other abnormalities that might interfere with nodule detection. In total, the dataset contains 1186 annotated nodules, which serve as reference for deep learning model training and evaluation.

The LUNA16 dataset incorporates images collected from seven academic centers and eight medical imaging companies, encompassing a diverse range of CT acquisition protocols and scanner models. The CT images were acquired using multiple scanner types, including GE Medical Systems LightSpeed scanners, Philips Brilliance scanners, Siemens Definition, Emotion, and Sensation scanner models, Toshiba Aquilion scanners.

This variation in image acquisition parameters ensures a heterogeneous dataset, improving the generalizability of deep learning models to real-world clinical scenarios.

**Fig. 2:**
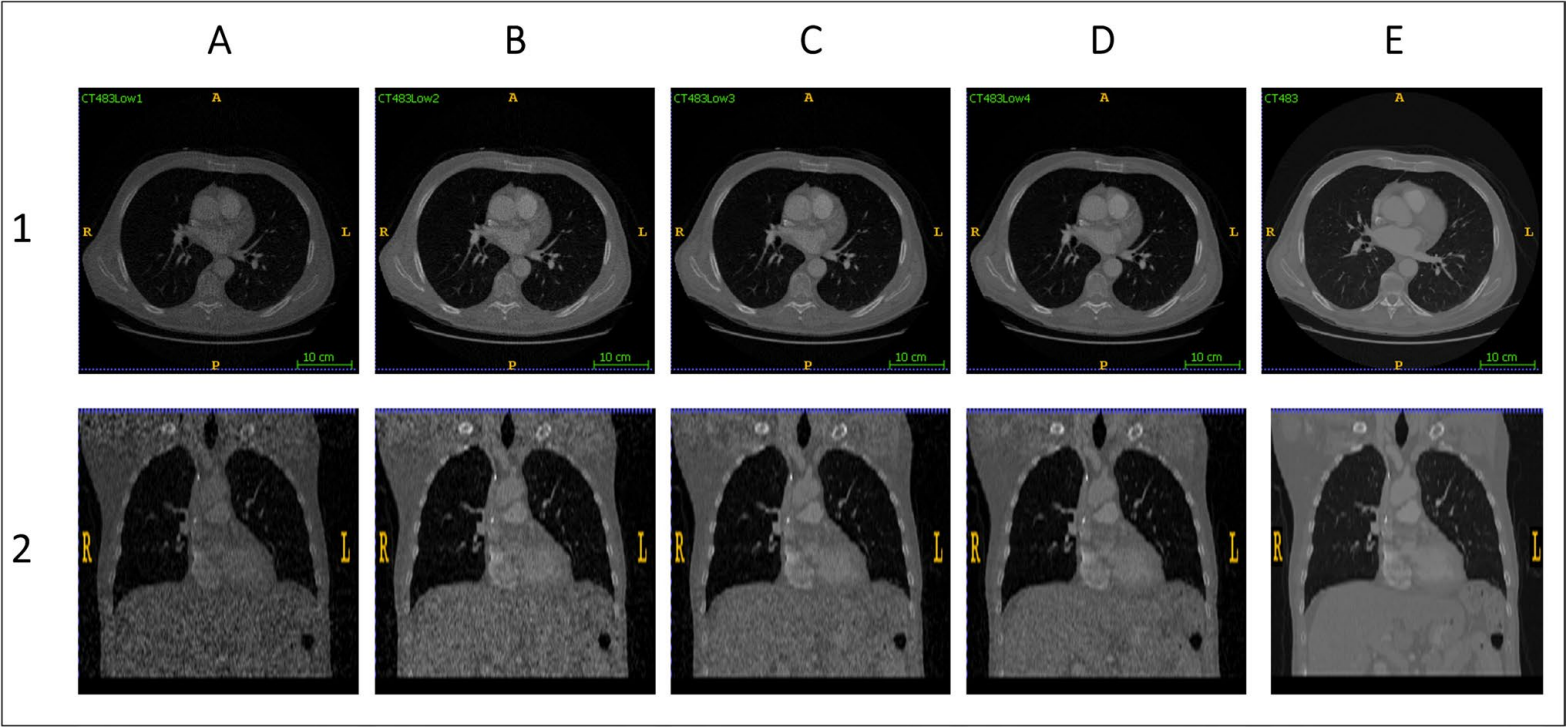
Transaxial and coronal views of **A** 10% low-dose, **B** 20% low-dose, **C** 40% low-dose, **D** 60% low-dose, and **E** full-dose (100%) CT images in the first and second rows, respectively

**Fig. 3:**
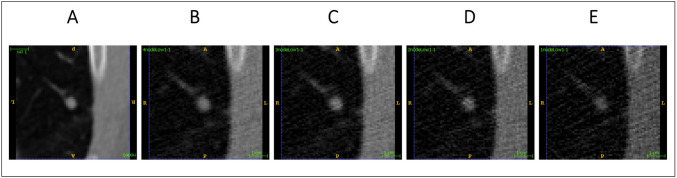
Transaxial view of 64 × 64 × 64 (voxel) patches extracted from **A** full-dose (100%), **B** 60% low-dose, **C** 40% low-dose, **D** 20% low-dose, and **E** 10% low-dose CT images depicting a suspicious nodule

CT images were resampled into an isotropic voxel size of 1 × 1 × 1 mm^3^. Then, 10%, 20%, 40%, and 60% low-dose versions were generated (more details in 2.3 section) from the full-dose CT images. The CT intensities were normalized to [0–1] range, which corresponds linearly to the Hounsfield Units (HUs). Figure 2 depicts representative slices of full-dose and low-dose CT images. Since the nodules are relatively small, the images should be cropped to eliminate background air and other irrelevant elements. To train and evaluate the models, matrices of 64 × 64 × 64 voxels were extracted from CT images (Fig. 3).

The dataset comprises 888 patient CT volumes, with 597 of them containing lung nodules, totaling 1126 nodules. From this pool, we chose 1000 nodules for our analysis, as some were excluded due to poor quality or incorrect annotation. Overall, we generated 1000 3D patches for nodules and 1000 3D patches for non-nodules, each with a size of 64 × 64 × 64.

To assess the generalizability of our proposed model, we used an external dataset. This dataset was gathered at Khatam’s PET/CT Center in Tehran and included 47 patients (37 males and 10 females) with an average age of 65.0 years (range: 41–85), presenting a total of 60 lung nodules and 60 non-nodules. The lesions were annotated using the same approach applied to LIDC-IDRI dataset. All subjects underwent 18 F-FDG PET/CT scans on a Biograph mCT scanner (Siemens Healthcare) equipped with 128 slices CT. A LDCT scan was acquired using the Siemens CARE Dose package, incorporating automatic exposure control (AEC) to maintain the lowest feasible patient dose for all examinations. The scanning parameters included a tube voltage set at 120 kVp, an effective tube current of 80 mA, a pitch of 0.8, and a reconstructed slice thickness of 2 mm. Despite the confirmation of lung nodules through PET data, the evaluation focused solely on CT images. The same preprocessing steps were also performed on this dataset.

### Low-dose CT image generation

Given that the noise in CT typically follows Poisson characteristics manifested during the acquisition process from different views, it is essential to introduce this noise in the projection space and then reconstruct the image. This process naturally results in a noisy image. LDCT images with 10%, 20%, 40%, and 60% of the full-dose scan were generated from the reference images (100% dose) using a simulation technique proposed by Wang et al. [34]. Based on Beer-Lambert’s equation (Eq. 1), the number of photons is diminished when they pass through an object:

1 $$Rf=-\text{ln}(\frac{{I}_{0}}{I})$$

where *R* is the Radon transform and *I*_0_ and *I* denote the initial number of photons and the incident number of photons impinging on the detector, respectively.

Additionally, the Radon transform is an integral transformation that generates projection data from a distribution map at a specific angle (Eq. 2).

2 $$Rf\equiv Rf(\rho ,\theta )={\int }_{-\infty }^{+\infty }f(\rho \text{cos}\theta -{\ell}\text{sin}\theta ,\rho \text{sin}\theta +{\ell}\text{cos}\theta )d{\ell}$$

where $\rho$ is the distance of an object from the center, $\theta$ is its angle in the Polar coordinate system, and *d* ${\ell}$ = *dxdy*.

To generate LDCT images, Poisson noise was applied to the projections (*Rf*) as follows:

1. The CT numbers were calculated from the full-dose CT images:
  3 $$HU=(\begin{array}{cc}Pixel& value\times Slope\end{array})+Intercept$$
  where *Slope* and *Intercept* are obtained from the image headers.
2. Owing to the standard voltage applied in CT imaging, *µ*_*air*_ = 0 was assumed.
3. Linear attenuation coefficients v calculated using Eq. 3.
  4 $${\mu }_{tissue}=(HU\times {\mu }_{water}/1000)+{\mu }_{water}$$
4. Projection data (*p*) were generated from the full-dose CT images using the Radon transform. Afterward, linear attenuation coefficients were derived from the projections.
5. The full-dose transmission (data are obtained from (Eq. 4)).
  5 $${T}_{fd}={e}^{-p}$$
6. The low-dose transmission data are obtained by applying Poisson noise to the standard data (Eq. 5).
  6 $${T}_{ld}=poisson({I}_{0}\times {T}_{fd})$$
7. Equation 6 was employed to calculate the low-dose projection data.
  7 $${P}_{ld}=\text{ln}(\frac{{I}_{0}}{{T}_{ld}})$$
8. Equation 3was used to convert the attenuation coefficients to CT values.
9. The low-dose images are obtained through filtered backprojection reconstruction.

According to Wang’s approach, we assumed *I*_0_ = 10000 as reference. LDCT images were generated by changing the number of received photons, for instance, *I*_0_ = 1000 for 10% low-dose level. It should be noted that conventional LDCT images are considered full-dose images, while the other levels are categorized as lower than LDCT or ultra-low-dose images.

### Model development

#### *Repeatingblocks network (RBNet)*

This study proposed a deep learning architecture, referred to as RBNet (repeating blocks network), a 3D convolutional neural network for the classification task. Regarding the developed classifiers, various approaches have been employed to enhance feature extraction and address associated challenges in analyzing these features. For instance, ResNet18 [38] introduced residual blocks to improve performance. MobileNet [39] utilized depthwise separable convolutions, while VGG16 [40] and AlexNet [41] employed multiple dense layers, resulting in a high number of trainable parameters.

There are four blocks in our model, each incorporating 3D convolution, 3D maxpooling, and batch normalization. The sequence of repeated blocks would lead to better feature extraction at different levels of complexity. This setting is able to extract features at four different levels per sequence, wherein maxpooling layers are inserted to reduce the dimensions of the image and highlight the discriminative features. Moreover, the issue of internal covariate shifts can be resolved by batch normalization. To mitigate the risk of overfitting in the final models, this approach incorporates dropout layers and global average pooling. These techniques aid in improving generalization and preventing the model from solely relying on specific features. By including these mechanisms, the data can be smoothly passed between intermediate layers, promoting better information flow and reducing the risk of overfitting. The integration of dropout layers and global average pooling enhances the model's ability to generalize well to unseen data, thus improving its overall performance and robustness. ReLu activation function was used for the internal layers, whereas a dense fully connected layer with the sigmoid function was considered in the final layer (generating labels of zero for non-nodules and one for nodules). Furthermore, increasing the number of CNN filters in each block of the 3D CNN classifier, from 64 in the first block to 256 in the last block, carries a number of advantages, including improved feature capturing capability, enhanced discrimination of patterns and structures, and the ability to learn hierarchical representations from the input data (Supplemental Fig. 1). It should be noted that our emphasis was on proposing a lightweight model compared to others, with a primary focus on CNN filters. These filters have proven to be exceptionally compatible with the task of image classification, allowing for efficiency without compromising performance.

#### Training full-dose model

The training process involved randomly selecting 60% of the data, comprising 1200 volumes (3D patches) that included 600 nodules and 600 non-nodules. To improve the accuracy of lesion classification, after hyperparameter tuning, a learning rate of 0.0001 was utilized with a decay factor of 0.96 to train the 3D convolutional model. Binary cross entropy was used as the loss function together with the Adam optimizer. To optimize the model's performance, we performed a grid search, testing different learning rates (0.00001 to 0.1), decay factors (0.1 to 0.99), and configurations of filter sizes and layers. We explored filter sizes ranging from 3 × 3 to 7 × 7, number of filters from 16 to 512, and different depths for the convolutional layers (3 to 9 layers). The best configuration corresponded to the lowest validation loss. The model was trained five times using full-dose images and different levels of low-dose images over about 40 epochs. Moreover, four other models (ResNet18, MobileNet, AlexNet, and VGG16) were developed and hyperparameter tuning was performed for finding the most efficient parameters on the same dataset (to be compared to our proposed model).

#### Training low-dose models

In this part, we exclusively trained the RBNet model using four distinct dose levels of CT images, specifically at 10%, 20%, 40%, and 60% dose levels.

#### Training the ensemble model (ERBNet)

To achieve a robust and adaptable model for classifying nodules and non-nodules in a dataset comprising various dose levels, we have introduced an ensemble weighted approach. We trained five models on distinct dose levels and combined them to create the Ensembled Repeating Block Network (ERBNet). To determine the optimal weights for each model in the ensemble, we employed the Dirichlet random function to generate 10,000 random weights and selected the most effective ones.

### Model evaluation

#### Full-dose evaluation

To assess the performance of various classifiers on full-dose CT images, we initially evaluated them on 400 nodules and 400 non-nodules. Finally, we applied the *t*-test method to calculate *P*-values for different classifiers compared to RBNet.

#### Low-dose evaluation

Independent models were developed (on RBNet) for full-dose and LDCT images. First, the model trained using full-dose CT images was evaluated using low-dose and full-dose CT images, and the corresponding results separately reported. Subsequently, the LDCT images were input to the corresponding models trained with the same low-dose levels (to assess the impact of developing dedicated models for each low-dose level). To validate the models, 400 nodules and 400 non-nodules were randomly selected and fed into the different models. The following metrics were employed to evaluate the different models.

#### ERBNet evaluation

In this particular phase, we evaluated the ERBNet model on a dataset containing 2000 nodules and non-nodules with 400 nodules for each dose level.

8 $$\begin{array}{cc}\text{Sensitivity}&SPC=True\;positive/\left(True\;positive+False\;negative\right)\end{array}$$

9 $$\begin{array}{cc}\text{Specificity}&SENS=True\;negative/\left(False\;positive+True\;negative\right)\end{array}$$

10 $$\begin{array}{cc}\text{Precision}&PPV=True\;positive/\left(True\;positive+False\;positive\right)\end{array}$$

11 $$\begin{array}{cc}\text{Accuracy}&ACC=\left(True\;positive+True\;negative\right)\end{array}/\left(Total\;decisions\right)$$

12 $$\begin{array}{cc}\text{F}1\text{ score}& F1=2\times \left(PPV\times SENS\right)\end{array}/\left(PPV+SENS\right)$$

where

13 $$Total\;decisions=True\;negative+True\;positive+False\;negative+False\;positive$$

Furthermore, receiver operating curves (ROC) were plotted for nodule classification using the different models. The ROC curve illustrates the true positive rate (sensitivity) versus the false positive rate (1 − specificity) at different levels of the binary classification threshold.

#### Evaluation of ERBNet on external dataset

In our external dataset, which includes 60 nodules and 60 non-nodules, we generated 24 CT volumes (comprising 12 nodules and 12 non-nodules patches) for each dose level. Subsequently, we evaluated the performance of our ensemble model using these volumes. It should be noted that all evaluations were conducted on a per-lesion basis, ensuring a detailed and specific evaluation for each individual lesion.

## Results

The performance analysis of different classifiers on the full-dose data revealed that RBNet exhibits superior results. RBNet outperforms other classifiers in terms of accuracy and sensitivity, demonstrating its superiority in these metrics. Additionally, RBNet achieves precision and specificity levels that are very close to those of ResNet18, albeit slightly lower.

**Fig. 4:**
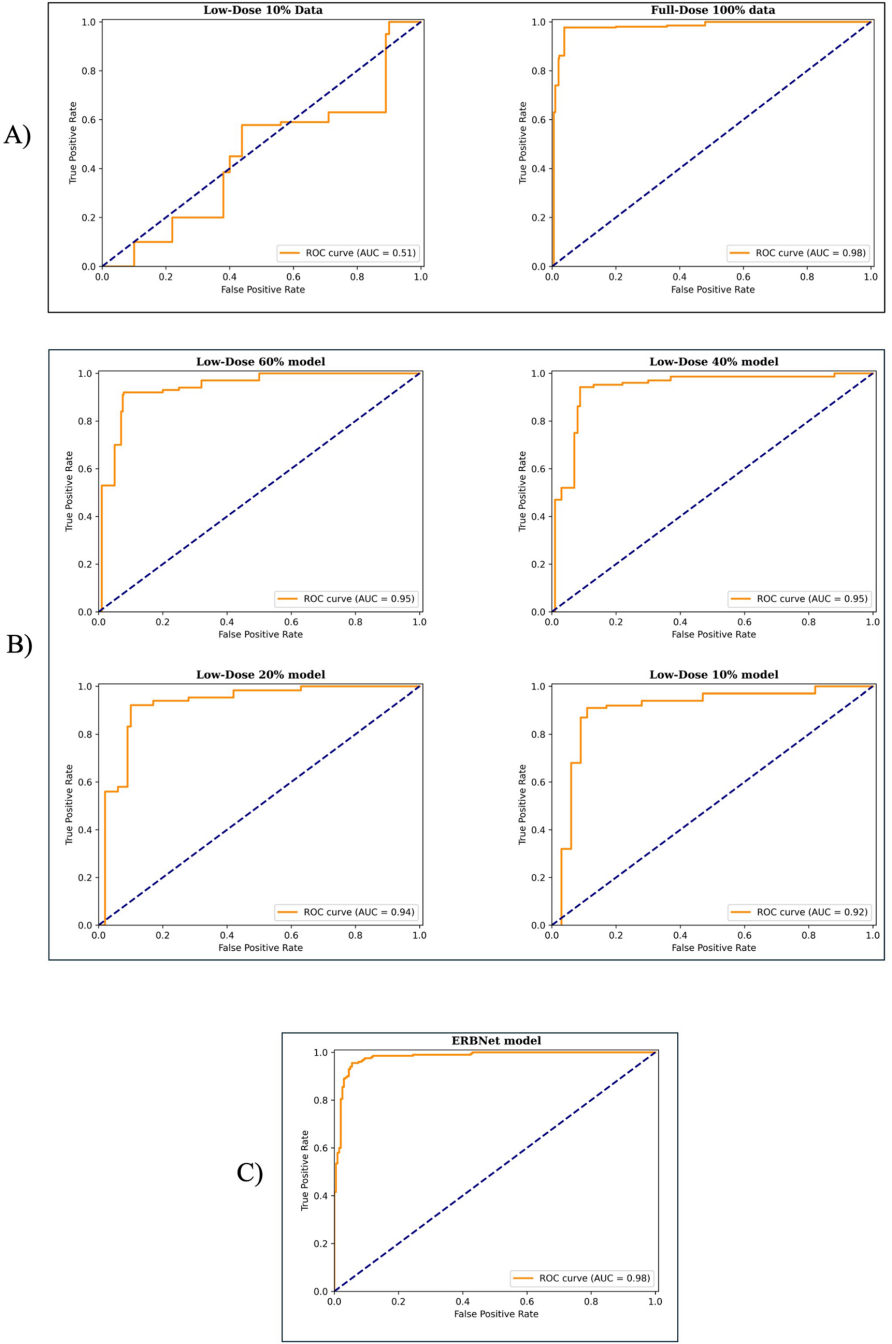
ROC plots summarizing model performance: (A) performance of the full-dose model evaluated on both low-dose (left) and full-dose (right) CT images; (B) nodule classification performance for low-dose models at 60% (upper left), 40% (upper right), 20% (lower left), and 10% (lower right) dose levels on the corresponding dose level; and (C) the ROC curve for the ERBNet model

The full-dose model resulted in an accuracy of 97.0% when evaluated on full-dose CT images. However, this model exhibited very poor performance on low-dose images as shown in Fig. 4 in terms of ROC metric. Table 1 presents the evaluation results of different classifiers. When the models were trained and evaluated using the same low-dose images, the lesion classification performance improved dramatically as reported in Table 2.

**Table 1:** Summary of model performance of different classifiers on full-dose images

Model	Accuracy	Sensitivity	Specificity	Precision	*P*-value (comparison with RBNet)
ResNet18	95.9%	95.0%	**97.0%**	**97.2%**	0.0230
MobileNet	96.7%	97.3%	96.0%	96.4%	0.0010
AlexNet	87.3%	83.9%	86.0%	91.2%	< 0.0001
VGG16	75.0%	62.3%	89.0%	86.0%	< 0.0001
RBNet	**97.0%**	**97.7%**	96.2%	96.3%	–

**Table 2:** Nodule diagnosis performance of low-dose and full-dose models

Data dose levels				Low-dose network						Full-dose network		
	Accuracy	AUC	Sensitivity	Specificity	Precision	F1 score	Accuracy	AUC	Sensitivity	Specificity	Precision	F1 score
LD 10%	90.0%	92.4%	91.0%	89.0%	89.2%	89.9%	60.0%	50.6%	60.2%	59.7%	59.9%	59.9%
LD 20%	91.1%	94.1%	92.2%	90.0%	90.2%	91.1%	60.0%	51.2%	60.7%	59.2%	59.8%	60.2%
LD 40%	92.7%	95.3.%	94.2%	91.25%	91.5%	92.8%	62.5%	51.7%	63.0%	62.0%	62.3%	62.6%
LD 60%	93.8%	95.7%	95.7%	92.2%	92.5%	93.9%	63.0%	50.2%	63.7%	62.2%	62.8%	63.2%
FD 100% (processed by the 10% model)	57.5%	51.3%	57.8%	56.2%	57.3%	57.6%	97.0%	98.2%	97.7%	96.2%	96.3%	97.0%

Tables 2 and summarizes the area under the curve (AUC) values for different models, showing that the model trained on full-dose images achieved an AUC of 98.2%, while models trained on lower dose images (FD 10%, FD 20%, FD 40%, and FD 60%) achieved AUCs of 92.4%, 94.1%, 95.3%, and 95.7%, respectively.

Ultimately, the evaluation of the ERBNet model on a combination of different dose levels in CT images yielded an accuracy of 95.0%, precision of 94.5%, sensitivity of 95.5%, specificity of 86.0%, F1 score of 94.9%, and AUC of 98.1%. These results indicate that the model is reliable for evaluating CT images acquired at various dose levels. Moreover, after evaluating the ensemble model with the in-house external dataset, we achieved an accuracy of 85.8%, precision of 83.0%, sensitivity of 90.0%, specificity of 81.6%, F1 score of 86.4%, and AUC of 85.4% (Table 3). Furthermore, Fig. 4 illustrates the ROC curves associated with the models’ performance.

**Table 3:** Performance evaluation of the ERBNet model on CT images at different dose levels and an in-house external dataset

Metric		Dataset
	Unseen test set (LUNA 16)	In-house external dataset (Khatam)
Accuracy	95.0%	85.5%
Precision	94.5%	83.0%
Sensitivity	95.5%	90.0%
Specificity	86.0%	81.6%
F1 score	94.9%	86.4%
AUC	98.1%	85.4%

## Discussion

The sensitivity decreased from 95.7 to 91.0% when the dose levels were reduced from 60 to 10% (Table 2). However, the sensitivity of the full-dose network evaluated using the full-dose network was 97.7% which could be regarded as a baseline to relatively assess other low-dose models. Increased noise levels in low-dose imaging would limit the performance of lesion classification models since the signal-to-noise ratio would be too low for some nodules and some noise-induced structures would be regarded as genuine nodules. Additionally, when assessing the ensemble model with the external dataset, the accuracy experiences a decrease from 95.0 to 85.8%. This indicates the need for further training with a more diverse range of data, including samples from different centers.

In comparison to our method focusing on conventional LDCT, the Squeeze-and-Excitation Vision Transformer (SE-ViT) proposed by [26], which integrates self-attention and squeeze-and-excitation mechanisms for enhanced feature recalibration, achieved an accuracy of 86.30%. The 4D U-Net architecture (combination of spatial and temporal information) leverages 3D lung nodule images for classification, achieving an accuracy of 92.84%. SAACNet [28], a multi-scale and channel-based feature extraction model, improved diagnostic performance with an accuracy of 95.18%. The fusion model proposed by Ma et al. [25] integrates radiomics and graph convolutional network features with deep CNNs for better nodule differentiation resulting in an accuracy of 92.18%. Lastly, Wu et al.’s [27] Self-Supervised Transfer Learning with Visual Attention (STLF-VA) framework addresses data scarcity by using self-supervised learning for robust nodule classification, achieving an accuracy of 92.36%. Recent models have become more complex due to the use of 3D patches, which often necessitate more sophisticated architectures. However, accuracy remains in the 90–97% range, similar to older studies [30, 31]. The complexity of these models can lead to overfitting, especially when the model's capacity exceeds the available data, and 3D patches of 64 × 64 × 64 size are still relatively small, causing complex models to overfit when using small patches. Additionally, these models are computationally expensive, requiring significant time and resources which is a crucial challenge in deep learning [35]. To address these challenges, we opted for a lightweight model that produces comparable results while being more efficient and cost-effective resulting in an accuracy of 97.0%. All metrics and comparisons are shown in Table 4. However, the main challenge occurs when image quality significantly changes due to variations in radiation dose, scanners, and protocols, resulting in a dramatic drop in model’s performance, as we observed a sensitivity decrease from 97.7 to 60.2% in our model when the LDCT radiation dose was reduced from 100 to 10% (Table 2).

**Table 4:** Comparison of the proposed model with previous works for nodules classification from standard-dose LDCT images

	Sensitivity	Specificity	Precision	F1 score	Accuracy	AUC
Ma et al. [21]	87.9%	94.8%	91.8%	89.7%	92.1%	94.8%
Xue et al. [22]	87.6%	–	87.2%	87.2%	86.3%	86.2%
Wu et al. [23]	91.6%	93.0%	98.0%	92.2%	92.4%	97.2%
Gu et al. [24]	97.3%	90.4%	–	–	95.2%	97.7%
Balci et al. [25]	92.4%	-	92.6%	92.5%	92.8%	96.2%
Ali et al. [26]	96.0%	97.4%	–	–	96.6%	99.1%
Halder et al. [27]	96.8%	95.2%	–	–	96.1%	99.4%
RBNet (proposed model)	97.7%	96.2%	96.3%	97.0%	97.0%	98.2%

In addition to training dedicated low-dose models, noise suppression models could be developed to estimate full-dose CT data from the low-dose versions. Then the model trained on the full-dose images can be applied to the synthetic conventional LDCT images for nodule classification. It can be concluded that denoising can be a useful approach for handling noisy images. This approach warrants further investigation and its performance should be compared with the models directly developed on low-dose images (the models evaluated in this work).

Table 2 reveals that both types of networks (low-dose and full-dose) have a better performance for identifying nodules rather than non-nodules. Specificity illustrates the performance of the networks for identifying non-nodules whereas the sensitivity does the same for nodules, as we assumed 0 for non-nodules and 1 for nodules. The similarity of many non-nodule structures in the lung, such as vessels, external particles, fat nodules, and condensed tissues to the nodules, challenges the identification and classification of the engaged nodules.

Regarding the model trained with conventional LDCT images in this work, a comparable performance was observed to state-of-the-art approaches reported in the literature. This model also showed promising robustness to increased noise levels where the accuracy of the model reduced from 97.0 to 90.0% when CT radiation doses reduced from 100 to 10%. The model trained with low-dose images demonstrated that lung nodule classification can be achieved on LDCT images without performing a denoising process, which may cause some sort of signal loss.

The ensemble model achieved an accuracy of 95.0%, indicating its ability to correctly classify the input samples. It achieved a precision of 94.5% and a sensitivity of 95.5%, reflecting its capability to accurately identify positive samples (nodules). Overall, the ERBNet model showed promising performance in classifying nodules and non-nodules, with high accuracy and sensitivity, while maintaining a reasonable level of precision and specificity. In addition, the ERBNet model demonstrated its capability to accurately classify lesions from LDCT images acquired at different dose levels. This ability makes it a valuable tool in medical imaging, as it provides reliable results across a range of dose levels, allowing for consistent and accurate assessment of pulmonary nodules.

The limitations of our study include the restricted quantity of appropriate LDCT images. Enhancing the dataset by incorporating images from diverse centers could bolster the robustness and precision of the model. Additionally, a noteworthy limitation of our study is the absence of access to authentic lower dose LDCT images, necessitating the utilization of simulated data. The implementation of a genuine low-dose imaging protocol would enhance the robustness and credibility of this investigation. Another limitation of this study is that the dataset lacks annotations on COPD, inflammation, and other related factors, which could potentially influence the performance of the deep learning model and its generalizability across different clinical conditions.

## Conclusions

In this study, we investigated the accuracy of lesion classification in LDCT imaging and to what extent increased noise levels in LDCT images would diminish the lesion classification accuracy. To this end, an algorithm based on convolutional networks (RBNet) was proposed to distinguish nodules and non-nodules from standard- and LDCT images. The model trained on full-dose CT images led to a lesion classification accuracy of 97.0%; however, its accuracy dropped dramatically when using LDCT images. The dedicated low-dose models (trained on LDCT images) led to promising lesion classification accuracy (> 90.0%). By developing an ensemble model using different models trained on various dose levels, we successfully achieved an accuracy of 95.0% for internal and 85.8% for external in-house dataset. This outcome highlights the effectiveness of ensemble approaches in handling non-homogeneous data with significant variability. This framework is a promising step for utilizing ultra-low dose CT images and can serve as a valuable tool for radiologists, offering assistance by allowing them to double-check their decisions on images with varying qualities, including those of very poor quality.

## Supplementary Information

Below is the link to the electronic supplementary material.

Supplementary file1 (PDF 134 KB)

## Data Availability

The data used in this study are publicly available from the Lung Nodule Analysis 2016 (LUNA16) dataset (*available at: *https://luna16.grand-challenge.org/Data/).

## Code availability

The code used in this work will be publicly available on GitHub upon publication of the paper.
