# Supplementary material for: Automated pulmonary nodule classification from low-dose CT images using ERBNet: an ensemble learning approach
Source: Med Biol Eng Comput. 2025 Apr 15;63(9):2767–79. doi: 10.1007/s11517-025-03358-2 (PMC12402046; doi:10.1007/s11517-025-03358-2)
Supplement: Supplementary file 1 — Supplementary file1 (PDF 134 KB) [file 11517_2025_3358_MOESM1_ESM.pdf]

Supplemental section

Introduction

Lung cancer is regarded as one of the major causes of cancer death worldwide [1]. For effective treatment and prevention of cancer progression, it is imperative to detect lung cancer at its earliest stages. Although the early representation of lung tumors/nodules is very subtle, early identification of lung cancer is feasible through meticulous imaging examinations. For early detection of lung cancer, it is crucial to identify the depicted nodules. However, many visible lung nodules are not malignant. CT imaging is considered to be one of the most effective methods for lung cancer detection at its early stages [2].

Regarding the classification of lung nodules and non-nodules on LDCT images, Ren et al. [3] proposed a method, referred to as MRC-DNN (Manifold Regularized Classification Deep Neural Network), to map 3D lung nodule patches to a low-dimensional manifold using a 3D CNN network. The manifold operation theoretically represents nodule characteristics, based on which malignant and benign nodules are classified. To this end, an encoder-decoder network was employed, achieving an accuracy, specificity, and sensitivity of 90%, 95%, and 81%, respectively, on the LIDC-IDRI (Lung Image Database Consortium and Image Database Resource Initiative) dataset [4]. Halder et al. [5] developed a two-pathway CNN model (2PMorpghCNN) to learn both the morphological and textural features of nodules. They achieved a sensitivity, specificity, and accuracy of 96.85%, 95.17%, and 96.10%, respectively, using the LIDC-IDRI dataset. Ali et al. [6] proposed a transferable texture convolutional neural network using an energy layer (EL) to extract texture features after three CNN layers. The EL decreases the number of learnable parameters to diminish the risk of over-fitting and model complexity. In another study, Zhang et al. [7] used an aggregated residual transformation (SE-ResNeXt) to classify malignant and benign nodules using the LUNA 16 dataset. It is worth emphasizing that the aforementioned studies focused solely on the full-dose or a single fixed-dose level. Therefore, further investigation is needed to evaluate LDCT images with various dose levels.

Neural network architecture

There are four blocks in the model, each incorporating 3D convolution, 3D maxpooling, and batch normalization (Supplemental Figure 1). The sequence of repeated blocks would lead to better feature extraction at different levels of complexity. This setting is able to extract features at four different levels per sequence, wherein maxpooling layers are inserted to reduce the dimensions of the image and highlight the discriminative features. Moreover, the issue of internal covariate shifts can be resolved by batch normalization. To mitigate the risk of overfitting in the final models, this approach incorporates dropout layers and global average pooling. These techniques aid in improving generalization and

preventing the model from solely relying on specific features. By including these mechanisms, the data can be smoothly passed between intermediate layers, promoting better information flow and reducing the risk of overfitting. The integration of dropout layers and global average pooling enhances the model's ability to generalize well to unseen data, thus improving its overall performance and robustness. ReLu activation function was used for the internal layers, whereas a dense fully connected layer with the sigmoid function was considered in the final layer (generating labels of zero for non-nodes and one for nodes). Furthermore, increasing the number of CNN filters in each block of the 3D CNN classifier, from 64 in the first block to 256 in the last block, carries a number f advantages, including improved feature capturing capability, enhanced discrimination of patterns and structures, and the ability to learn hierarchical representations from the input data.

Discussion

During the first phase of this study, our objective was to create an efficient model for classifying pulmonary nodules and non-nodules. To achieve this, we proposed a relatively shallow network with a limited number of parameters. In addition, we trained four other widely recognized classifiers in order to compare their performance. The results indicated the superior performance of the RBNet model in terms of accuracy and sensitivity compared other classifiers (Table 1), while showing competitive specificity and sensitivity. RBNet's exponential decay learning rate contributed to its excellent performance that allowed the model to effectively adjust the learning rate during training, thus resulting in better convergence while potentially avoiding issues, such as getting stuck in suboptimal solutions. Furthermore, RBNet's advantage is the lower number of parameters which reduces computational cost while addressing the problem of high variance and overfitting that often arises in complex models. By leveraging CNN layers, RBNet effectively extracts features from different complexity levels, while techniques like dropout and global average pooling further enhance the model's generalization capabilities.

In the next phase, RBNet was trained using the full-dose CT images for nodule classification in the lung, and the model was evaluated with images at different dose levels (10%, 20%, 40%, 60%, and 100%). Since the performance of the model trained with full-dose CT images was very poor for low-dose images, dedicated models were developed for each low-dose level. These models were evaluated using the same LDCT images (using an unseen external dataset). Further evaluation of the 10% low-dose model was conducted using full-dose images. Regarding the dedicated low-dose models, the accuracy of lesion classification was remarkably enhanced compared to the full-dose model evaluated using the different LDCT images. Moreover, the 10% low-dose model exhibited poor performance when full-dose images were fed into this model for evaluation. Briefly, when the models were trained and evaluated with the same low-dose images (the same dose levels not the same samples), their performance for lesion classification improved remarkably.

In a study conducted by Wu et al. [8], the authors achieved a high sensitivity of 95% for the classification of malignant and benign nodules from full-dose images. However, their specificity was not promising (about 81%) for full-dose LIDC-IDRI images. Their network had a better performance to identify positive cases (nodules) rather than detecting non-nodules. Halder et al. [5] achieved a sensitivity of 96.85% and specificity of 95.17 % for both nodules and non-nodules using a 2-pathway CNN which has two different paths for extracting both textural (using CNN filters) and morphological (applying white tophat operation) features. These works trained and evaluated the models using standard-dose CT images (no evaluations on LDCT images were performed). In this regard, the full-dose model developed in this study exhibited a comparable or even superior performance compared to previous works, achieving a sensitivity of 97.7% and specificity of 96.2%. In comparison, Zhang et al. employed the SE-ResNeXt model, which is a modification of ResNet [7] and reported an accuracy of 91.6%. Ali et al. [6] achieved an accuracy of 96.69% using a combination of Energy layer and a CNN-based model for nodule/non-nodule classification from standard dose CT images as reflected in Table 4. It should be noted that their model involved 2,263,170 trainable parameters, whereas the RBNet model (proposed in this study) contained only 1,286,849 trainable parameters. An attention-based network proposed by Wu et al. [8] achieved an accuracy of 92%.

In a study by Huang et al. [9], a 3D CNN-based classification model was proposed for pulmonary nodule classification using image patches. The model incorporated attention mechanisms, multi-level feature fusion, and a hybrid loss, resulting in impressive performance metrics, including an accuracy of 85.3%, sensitivity of 86.8%, specificity of 83.9%, and an AUC of 0.9042. These findings demonstrate the enhanced diagnostic capability of the system for pulmonary nodules. However, it is important to note that attention-based models may encounter limitations when confronted with small datasets, as exemplified in this study with 400 malignant and 400 benign nodules from The National Lung Screening Trial (NLST) dataset [10]. Conversely, simpler 3D CNN-based models like RBNet require less data and often deliver more reliable outcomes in such scenarios. Considering the widespread scarcity of large datasets, optimizing model development to effectively handle limited data is essential for achieving improved performance.

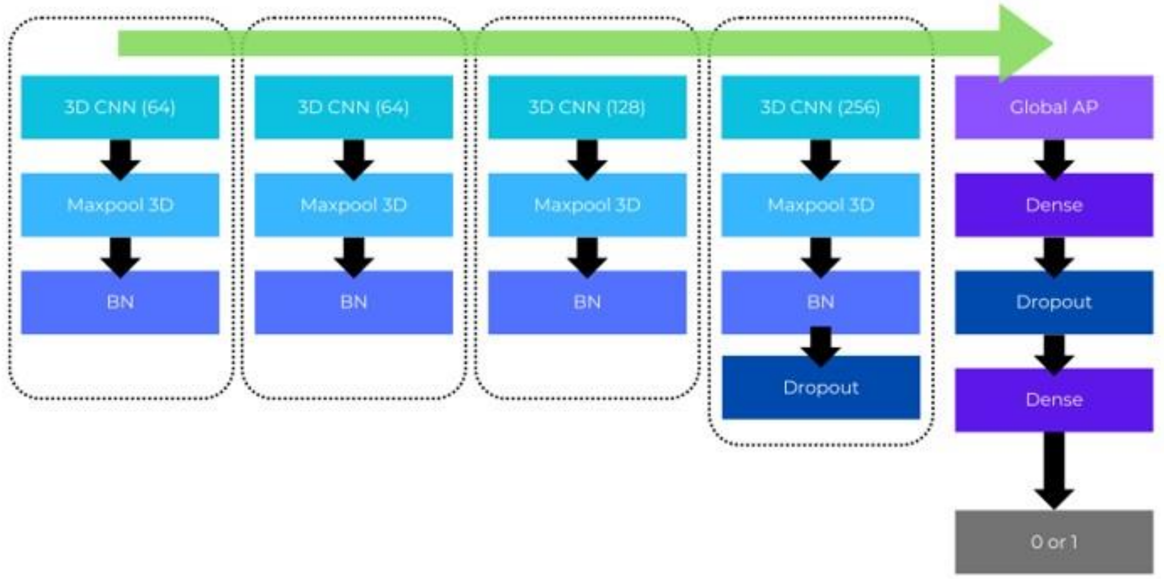

Supplemental Figure 1. Architecture of the proposed model for lung nodule classification.

References

1 Wood DE, Kazerooni EA, Aberle D et al (2022) NCCN Guidelines® Insights: Lung cancer screening, version 1.2022: Featured updates to the NCCN guidelines. Journal of the National Comprehensive Cancer Network 20:754-764

2 Gohagan J, Marcus P, Fagerstrom R et al (2004) Baseline findings of a randomized feasibility trial of lung cancer screening with spiral CT scan vs chest radiograph: the Lung Screening Study of the National Cancer Institute. Chest 126:114-121

3 Ren Y, Tsai M-Y, Chen L et al (2020) A manifold learning regularization approach to enhance 3D CT image-based lung nodule classification. International journal of computer assisted radiology and surgery 15:287-295

4 Armato SG, 3rd, McLennan G, Bidaut L et al (2011) The Lung Image Database Consortium (LIDC) and Image Database Resource Initiative (IDRI): a completed reference database of lung nodules on CT scans. Med Phys 38:915-931

5 Halder A, Chatterjee S, Dey D (2022) Adaptive morphology aided 2-pathway convolutional neural network for lung nodule classification. Biomedical Signal Processing and Control 72:103347

6 Ali I, Muzammil M, Haq IU, Khaliq AA, Abdullah S (2020) Efficient lung nodule classification using transferable texture convolutional neural network. Ieee Access 8:175859-175870

7 Zhang G, Yang Z, Gong L, Jiang S, Wang L, Zhang H (2020) Classification of lung nodules based on CT images using squeeze-and-excitation network and aggregated residual transformations. La radiologia medica 125:374-383

8 Wu R, Liang C, Li Y, Shi X, Zhang J, Huang H (2023) Self-supervised transfer learning framework driven by visual attention for benign–malignant lung nodule classification on chest CT. Expert Systems with Applications 215:119339

9 Huang Y-S, Wang T-C, Huang S-Z et al (2023) An improved 3-D attention CNN with hybrid loss and feature fusion for pulmonary nodule classification. Computer methods and programs in biomedicine 229:107278

10 Team NLSTR (2011) The national lung screening trial: overview and study design. Radiology 258:243-253
